# Supplementary material for: Causal Relationship between Aging and Anorexia Nervosa: A White-Matter-Microstructure-Mediated Mendelian Randomization Analysis
Source: Biomedicines. 2024 Aug 16;12(8):1874. doi: 10.3390/biomedicines12081874 (PMC11351342; doi:10.3390/biomedicines12081874)
Supplement: Supplementary file 1 [file biomedicines-12-01874-s001.zip › Supplementary Figure/Figure S4/Singe SNP plot of MR analysis for TL to ubm-b-2112.pdf]

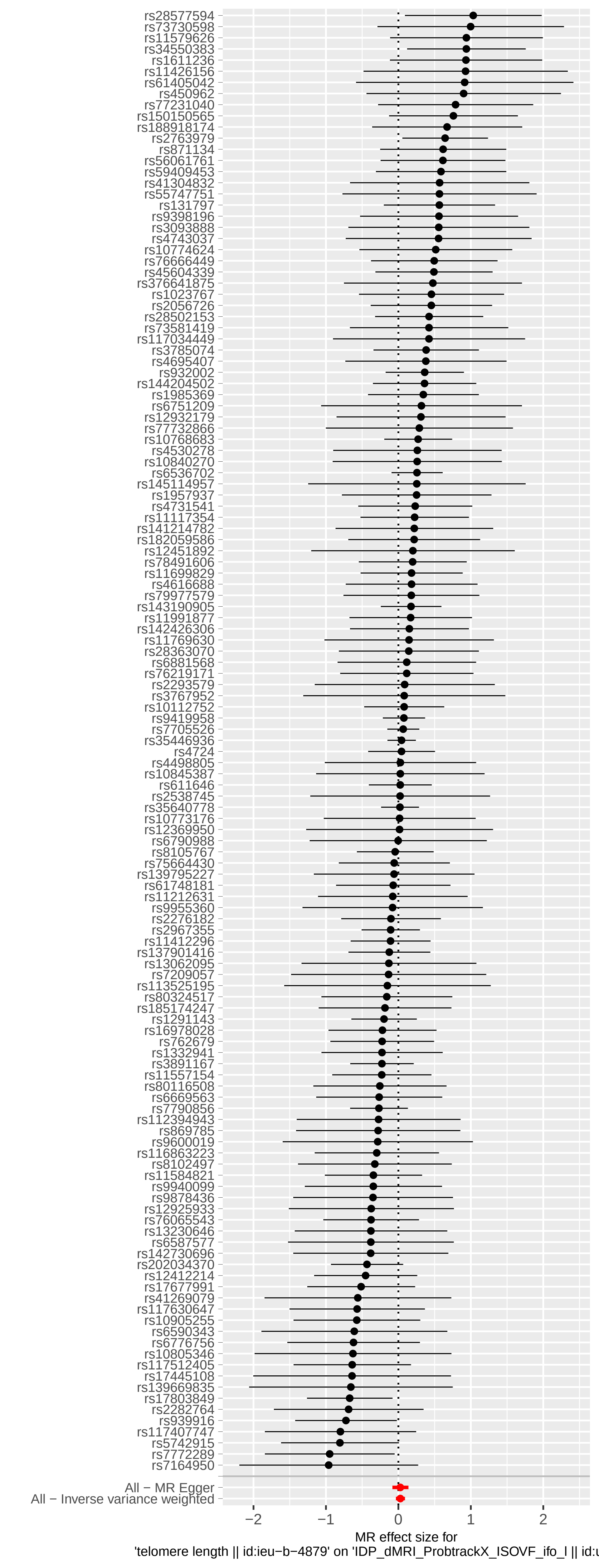
rs28577594
rs73730598
rs11579626
rs34550383
rs1611236
rs11426156
rs61405042
rs450962
rs77231040
rs150150565
rs188918174
rs2763979
rs871134
rs56061761
rs59409453
rs41304832
rs55747751
rs131797
rs9398196
rs3093888
rs4743037
rs10774624
rs76666449
rs45604339
rs376641875
rs1023767
rs2056726
rs28502153
rs73581419
rs117034449
rs3785074
rs4695407
rs932002
rs144204502
rs1985369
rs6751209
rs12932179
rs77732866
rs10768683
rs4530278
rs10840270
rs6536702
rs145114957
rs1957937
rs4731541
rs11117354
rs141214782
rs182059586
rs12451892
rs78491606
rs11699829
rs4616688
rs79977579
rs143190905
rs11991877
rs142426306
rs11769630
rs28363070
rs6881568
rs76219171
rs2293579
rs3767952
rs10112752
rs9419958
rs7705526
rs35446936
rs4724
rs4498805
rs10845387
rs611646
rs2538745
rs35640778
rs10773176
rs12369950
rs6790988
rs8105767
rs75664430
rs139795227
rs61748181
rs11212631
rs9955360
rs2276182
rs2967355
rs11412296
rs137901416
rs13062095
rs7209057
rs113525195
rs80324517
rs185174247
rs1291143
rs16978028
rs762679
rs1332941
rs3891167
rs11557154
rs80116508
rs6669563
rs7790856
rs112394943
rs869785
rs9600019
rs116863223
rs8102497
rs11584821
rs9940099
rs9878436
rs12925933
rs76065543
rs13230646
rs6587577
rs142730696
rs202034370
rs12412214
rs17677991
rs41269079
rs117630647
rs10905255
rs6590343
rs6776756
rs10805346
rs117512405
rs17445108
rs139669835
rs17803849
rs2282764
rs939916
rs117407747
rs5742915
rs7772289
rs7164950
All − MR Egger
All − Inverse variance weighted
−2
−1
0
1
2
MR effect size for
'telomere length || id:ieu−b−4879' on 'IDP_dMRI_ProbtrackX_ISOVF_ifo_l || id:u
